# Insight on Reaction Pathways of Photocatalytic CO_2_ Conversion

**DOI:** 10.1021/acscatal.2c01012

**Published:** 2022-06-03

**Authors:** Yiou Wang, Enqi Chen, Junwang Tang

**Affiliations:** † Department of Chemical Engineering, University College London, London WC1E 7JE, U.K.; ‡ Department of Physics, Ludwig-Maximilians-Universität München, Königinstr. 10, 80539 Munich, Germany

**Keywords:** CO_2_ conversion, photocatalysis, mechanism, reaction pathways, selectivity

## Abstract

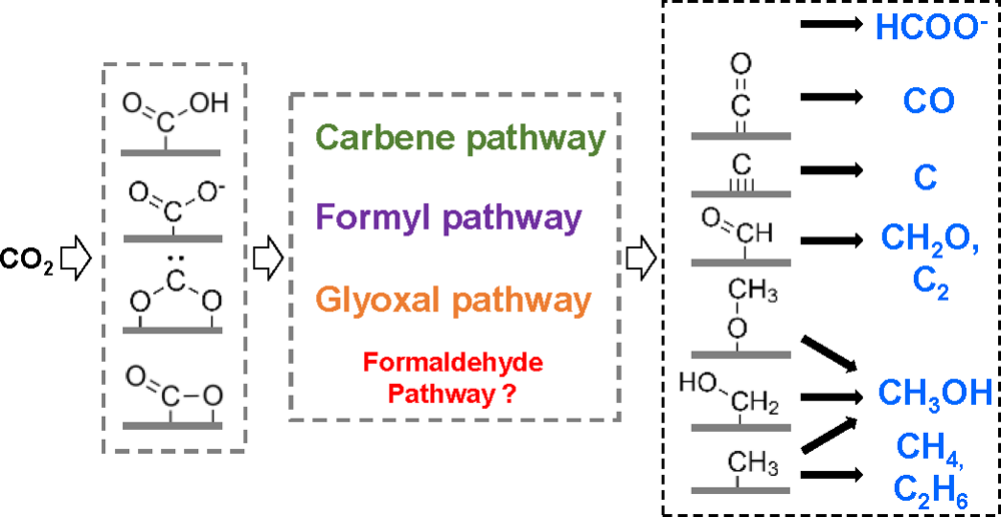

Photocatalytic CO_2_ conversion to value-added chemicals is a promising solution to mitigate the current energy and environmental issues but is a challenging process. The main obstacles include the inertness of CO_2_ molecule, the sluggish multi-electron process, the unfavorable thermodynamics, and the selectivity control to preferable products. Furthermore, the lack of fundamental understanding of the reaction pathways accounts for the very moderate performance in the field. Therefore, in this Perspective, we attempt to discuss the possible reaction mechanisms toward all C_1_ and C_2_ value-added products, taking into account the experimental evidence and theoretical calculation on the surface adsorption, proton and electron transfer, and products desorption. Finally, the remaining challenges in the field, including mechanistic understanding, reactor design, economic consideration, and potential solutions, are critically discussed by us.

## 1 Introduction

The atmospheric CO_2_ content has inevitably increased from 275 ppm to over 400 ppm since the industrial evolution, far exceeding its natural fluctuation (180–300 ppm) over the past 800 000 years because of an emission rate of more than 2 ppm per annum.^1,2^ The reactions emitting CO_2_, such as the combustion of fossil fuels, could directly release heat and, in most cases, also produce water that is also a greenhouse gas in its vapor form.^3^ The IR-active CO_2_ molecules trap the thermal radiation from the Earth’s surface and the sunlight, raising the global temperature and subsequently liberating even more CO_2_ by shifting the CO_2_-carbonate dissolution equilibrium in the oceans.^4^ Once the natural carbon-cycle capacity is overwhelmed, an adverse process leads to dramatic climate change and other environmental issues, highlighting the urgent need for artificial approaches to maintain the carbon balance by capturing CO_2_ for the carbon-neutral economy.^5^

**Scheme 1:**
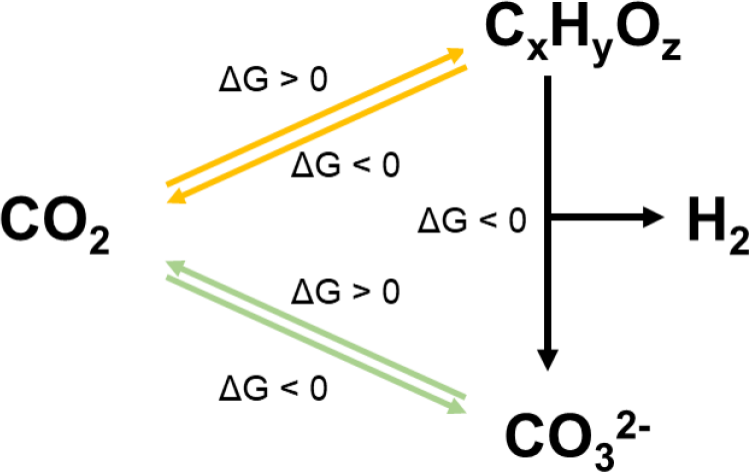
Illustration for the Natural Carbon Cycles Including CO_2_ to Organic and Inorganic Pathways

The carbon on Earth simply moves between different reservoirs because of geological and geochemical processes as well as human activities, while the total carbon amount has remained constant (Scheme 1).^6^ The new technology for CO_2_ utilization should at least be carbon-neutral either via exergonic pathways or endergonic ones but driven by renewable energy sources. Inspired by the fact that the majority of carbon on the Earth’s surface is stored in the form of carbonates, scientists have developed thermodynamically favorable CO_2_-to-carbonate mineralization technologies.^7^ Recently, the concept of converting a carbon-containing fuel into carbonates and carbon-free hydrogen was reported.^8^ Meanwhile, inspired by natural photosynthesis, scientists have also made significant progress in uphill reactions to fix CO_2_ into high-value fuels with the input of energy from sunlight. Such sustainable solar fuels are attractive with a satisfactory density of energy stored in their chemical bonds (*e.g.*, 20 MJ/kg for methanol), which are significantly higher than current batteries (∼0.1–0.7 MJ/kg) and able to be released on demand without additional carbon emission.^9−11^ The storage of almost inexhaustible solar energy in CO_2_-reduction fuels will significantly contribute to balance the carbon-cycle and address the issues of carbon-neutral energy supply,^11^ leading to the concept of the photocatalytic process for CO_2_ reduction.

The earliest demonstration of photoassisted CO_2_ reduction in water dates back to the late 1970s when the Pt-SrTiO_3_ was used to convert gaseous water and CO_2_ to methane, as reported by Hemminger et al., and many semiconductors (TiO_2_, ZnO, CdS, GaP, and SiC) were also shown to reduce aqueous CO_2_ to formic acid, formaldehyde, methanol, and methane by Inoue et al.^12,13^ Since then, many efforts have been devoted not only to improve the efficiency of CO_2_ photoconversion or the related but also to tune the selectivity of a specific product.^14,15^ Moreover, the formation of C_2+_ products has attracted emerging attention because of their high value compared with their C_1_ counterparts.^16,17^ Despite the advancement made in the past decades, the performance of CO_2_ photoconversion is still unsatisfactory, far from the target 10% solar-to-fuel efficiency, because of a few intrinsic reasons.

**Scheme 2:**
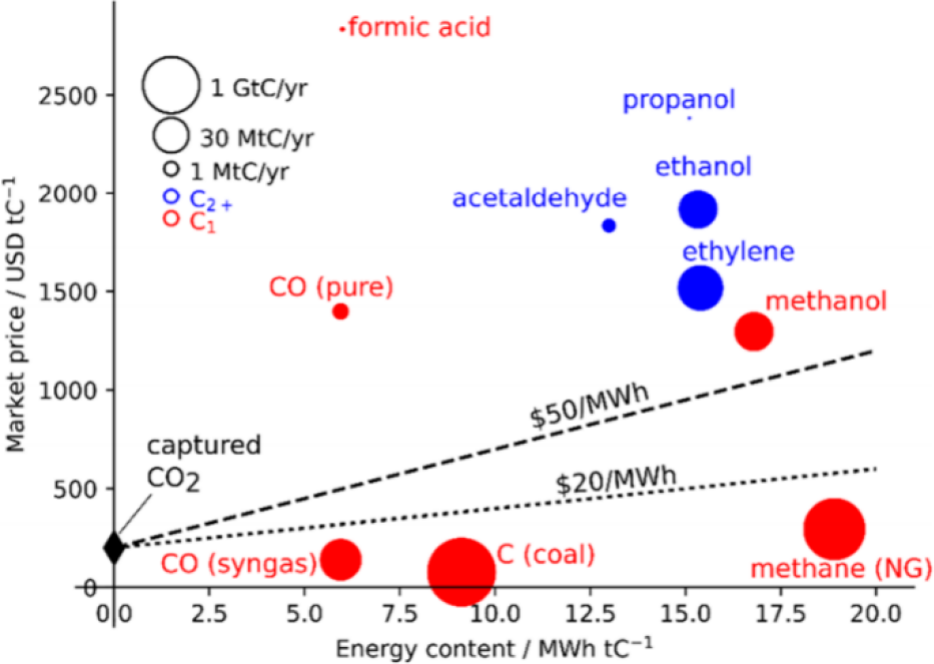
Market Price of CO_2_ Recycling Products as a Function of Energy Content. Reproduced from Ref (16). Copyright 2019 American Chemical Society Dotted lines represent minimum energy cost required for CO_2_ capture and conversion based on the present commercially available technology.

(1) The difficulty in activation of inert CO_2_. Owing to the closed-shell electronic configuration, linear geometry, and D_∞h_ symmetry,^18^ it requires −1.9 V potential (vs NHE at pH 7) to activate CO_2_ and to form an anion radical CO_2_^–^, which the majority of semiconductors could hardly provide except for a few reports (*e.g.*, poly *p*-phenylene in trimethylamine).^19−22^ A possible solution to lower the barrier to the reduction reaction is to adsorb CO_2_ on the surface of catalysts and then reduce CO_2_ together with protons, which usually requires the loading of active cocatalysts or the engineering of surficial defects such as oxygen vacancies.^19,20,23,24^
(2) The challenging multiple-electron kinetics. It is commonly believed that CO_2_ reduction is a process via multi-proton and multi-electron transfer, lowering the CO_2_ reduction potentials close to that of proton reduction, making it much more difficult than the two-electron proton reduction process to hydrogen.^17^ Except for some reports of molecular catalysts, it is challenging to validate whether the reactions proceed via a simultaneous multi-electron transport process or through a cascade of one-electron steps, where the first electron transfer seems to be the limiting step.^25,26^ Another question is whether the lifetime of the charge carriers matches that of the surface reactions of interest.
(3) Unfavorable thermodynamics of CO_2_ reduction by water. Opposite to the exothermal combustion of fuels, the production of solar fuels from CO_2_ and water is endothermal (*e.g.*, Gibbs free energy of 818.3 kJ/mol for CH_4_ and 702.2 kJ/mol for CH_3_OH).^27^ Although using hydrogen instead of water could turn CO_2_ reduction into exothermic reactions hence having demonstrated much-enhanced activity, these thermal catalytic processes usually proceed under higher temperatures (>150 °C) even promoted by light, requiring an additional step and energy for water electrolysis to hydrogen. Thus, hydrogenation systems and those using sacrificial hole scavengers will not be included in this Perspective as they are debatably sustainable to some extent. As the water oxidation half-reaction is very sluggish because of the four-hole chemistry and the likely oxidation of the reduction intermediates before the multi-electron carbon products are generated,^28^ using water to reduce CO_2_ to valuable chemicals is exceptionally challenging. In short, to overcome the obstacles mentioned above for CO_2_ photoreduction, besides the commonly existing issues for photocatalysis such as light-harvesting, one has to carefully tailor the photocatalysts, cocatalysts and the reaction conditions based on the fundamental understandings.
(4) Selectivity toward preferable products. Various products could be obtained from CO_2_ conversion while tuning the selectivity remains the major challenge. It should be noted that not all of the products are value-added chemicals if the cost of CO_2_ capture is taken into consideration (Scheme 2).^16^ For example, the commonly observed CO (with low purity) and CH_4_ are economically unfavorable due to their relatively low market price. The most profitable C_1_ product is formic acid, which unfortunately has a low energy content. Methanol and C_2+_ products are more attractive because of their outstanding market price, market demand, and energy content. However, because of the lack of molecular-level understanding of reaction mechanisms, the selectivity control to preferable products is still challenging.

**Scheme 3:**
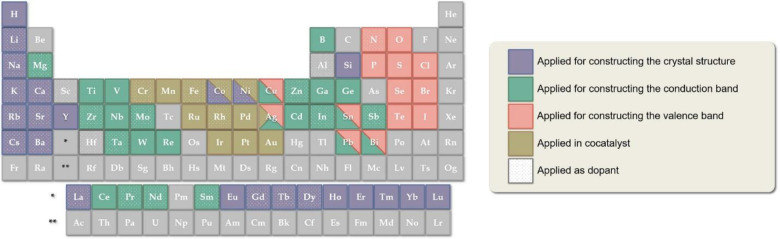
Elements Used to Construct Photocatalysts for Photocatalytic Water Splitting and Their Roles. Reproduced from Ref (32). Copyright 2019 American Chemical Society Alkali metals, alkaline earth metals, and some rare-earth ions usually do not directly contribute to the formation of bands and just construct crystals structure (*e.g.*, as the A position of cations in perovskite). Most metal oxide photocatalysts are composed of metal cations with d^0^ (Ti^4^ + , Zr^4+^, Nb^5+^, TA^5+^, V^5+^, W^6+^, and Ce^4+^) or d^10^ (Zn^2+^, In^3+^, Ga^3+^, Ge^4+^, Sn^4+^, and Sb^5+^) configurations. The conduction bands for d^0^ and d^10^ metal oxide photocatalysts typically consist of the d and sp orbitals of the metal cations, respectively. The valence band of semiconductors usually consists of the p orbitals of N, P, O, S, Se, Te, Cl, and so on. Cocatalysts elements include, for example, Cr, Mn, Fe, Ru, Rh, Pd, Ag, Ir, Pt, Au. Some elements have more than a single function.

Apart from the four key factors as mentioned above, the core of CO_2_ photoreduction lies in the selection of semiconductor photocatalysts. The progress of semiconductor photocatalyst development has been heavily summarized by many comprehensive reviews.^20,27,29−31^ Also, the strategies to design novel photocatalysts for CO_2_ reduction resemble those for water splitting, which could be found in broad literatures (Scheme 3).^32−34^ For example, Li et al. summarized the selectivity for photocatalytic reduction of CO_2_ over different cocatalysts.^31^ Briefly, Pd, Pt, or Au are more favorable for CH_4_ production; Ag for CO, CH_4_, or CH_3_OH; Cu for hydrocarbons; and Cu_2_O, RuO_2_, or NiO_*x*_ for CH_3_OH production. Therefore, complementary to these reviews on photocatalysts’ development, here we aim to discuss the possible reaction pathways and mechanisms on the basis of our understandings in this Perspective, including how to design photocatalysts and how to obtain this fundamental information by spectroscopies, as well as the principles for various products. Furthermore, we propose challenges facing this field, such as the characterizations needed to validate and understand the experimental process and pathways to practical applications.

## 2 REACTION PATHWAYS AND POSSIBLE PRODUCTS

**Scheme 4:**
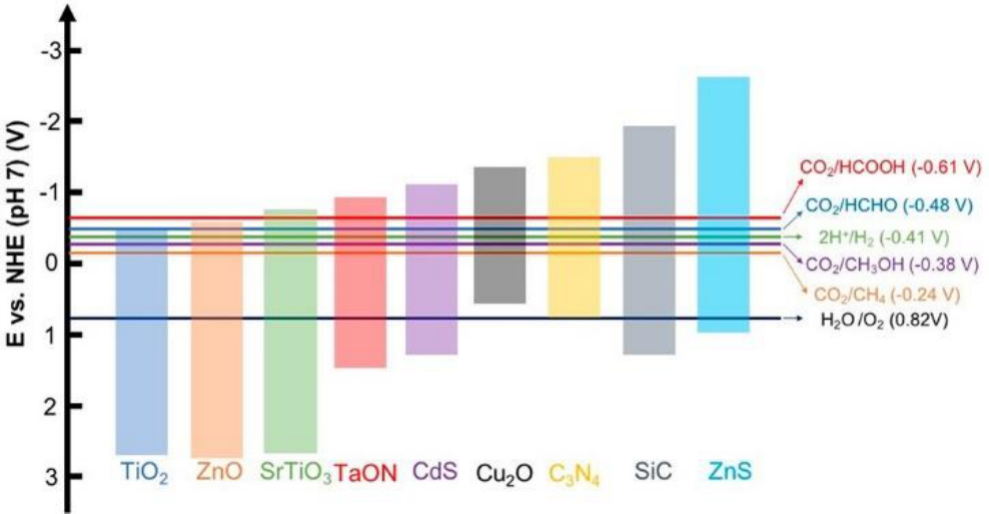
Band Alignments of a Few Representative Semiconductor Photocatalysts and the Related Redox Potentials to CO_2_ Reduction Reactions to Different Products. Reproduced with Permission from Ref (17). Copyright 2020 American Chemical Society

Scheme 4 lists the redox potential of various products from photocatalytic CO_2_ conversion and the band alignment of a few representative photocatalysts.^17^ To determine the selectivity and efficiency of the process, gas chromatography coupled with mass spectrometry (GCMS) is commonly used to quantify the products, while for nonvolatile ionic formate or oxalate products, ion-exchange chromatography is necessary. Understanding the exact reaction mechanism of CO_2_ photoreduction relies on evidence from experimental and theoretical methods. Infrared spectroscopy (IR) is helpful for surface chemistry studies, and electron paramagnetic resonance (EPR) is an essential tool to reveal the mechanism via the detection of paramagnetic unpaired electrons of the intermediates. The charge carrier dynamics could be analyzed by transient absorption measurements, together with modeling. Important information regarding adsorption and kinetics could also be provided by electrochemical measurements and theoretical computation.

**Scheme 5:**
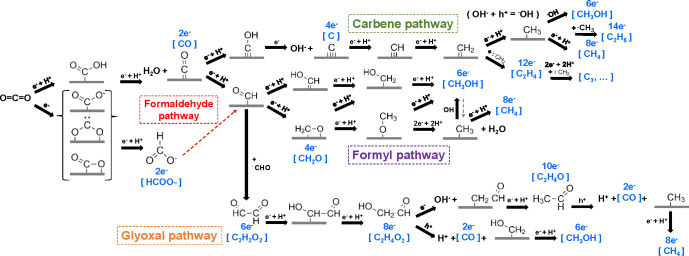
Mechanistic Pathways of CO_2_ Reduction to Commonly Observed C_1_ and C_2_ Products

In an earlier review, Habisreutinger et al. had summarized three possible mechanisms for the photocatalytic formation of five C_1_ (CO, HCOOH, CH_2_O, CH_3_OH, CH_4_) and some C_2_ products from CO_2_ on TiO_2_, namely the formaldehyde pathway, the carbene pathway, and the glyoxal pathway.^14^ These different possibilities start with the binding modes of CO_2_ on the surface of catalysts, including oxygen coordination, carbon coordination, and side/mixed coordination.^36^ On the basis of these, we proposed a comprehensive description of pathways (Scheme 5). The pathways are directed by whether the following reaction occurs via electron transfer, proton transfer (sometimes hydroxyl transfer) or concerted electron–proton transfer.^16^ An intermediate molecule in one pathway could be the final product of another pathway if it fast desorbs from the surface of the catalysts before further steps take place. Since the final products from the experiments are the most direct clues, we thus discuss the possible mechanistic details guided by respective products and the issues open to be elucidated.

As mentioned above, using water to reduce CO_2_ is the ideal and sustainable process, which includes two half-reactions, water oxidation (eq 1) and multi-electron reduction of CO_2_. The following details all these reduction processes, involving the production of C_1_ (*e.g.*, formic acid, carbon monoxide, formaldehyde, carbon, methanol, and methane) and C_2_ products (*e.g.*, oxalic acid, acetic acid, acetaldehyde, ethanol, ethylene, ethane) by photocatalysis.
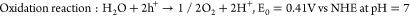
1

### Pathways to C_1_ Products

#### Two-Electron Reduction Process

The two-electron reduction process produces CO and HCOOH on the basis of eqs 2–5, and HCOOH production happens together with water oxidation (eq 1).
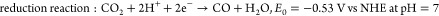
2
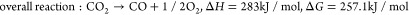
3
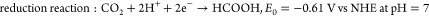
4
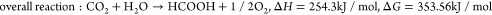
5

Carbon monoxide plays a crucial role in the Fischer–Tropsch synthesis and carbonylation of alkenes, while formic acid is a necessary preservative and antibacterial agent in livestock feed.^17^ Regarding the numbers of electrons needed, CO and HCOOH are kinetically the most accessible products from CO_2_, while thermodynamically, both are uphill reactions. Meanwhile, it should be noted that the minimal potential for CO_2_ reduction decreases with the increase of the number of electrons involved in the products. For example, photocatalytic CO_2_ conversion to CO or HCOOH requires slightly more negative potential than that to CH_4_.

CO, as shown in Scheme 5, is a product, a byproduct or an intermediate in CO_2_ reduction. In detail, CO is an intermediate in the carbene pathway after anchoring the C atom of CO_2_ to the surface of the catalysts. One proton attacks the O atom in CO_2_ to form COOH radicals in the presence of the first electron, followed by an immediate cleavage of the C–OH bond, which releases CO and water if not proceeding further.^37,38^ The selectivity of this process could be controlled by the binding strength of CO on the surface of a catalyst. CO can be a poison if it binds too strongly to the metal, while CO desorbs as a final product before further reduction occurs if the binding is too weak.^39^ Studies have shown that the suitable binding energy of CO on the copper catalyst and the high coverage ensures the formation of more reduced products and inhibits the competitive hydrogen evolution.^40^ In the glyoxal pathway, CO is a byproduct when the C_2_ intermediates decarbonylize to form methanol or methane,^41^ which agrees well with the literature that CO is commonly detected together with methanol and methane products. The oxidation of organic products such as formaldehyde and methanol by photoholes also results in CO, which we observed.^42^ Such backward reactions convert the kinetically more challenging reduction products to a two-electron product CO, which can be mitigated by hindering the adsorption of the formaldehyde and methanol on the oxidation sites.^42,43^

**Scheme 6:**
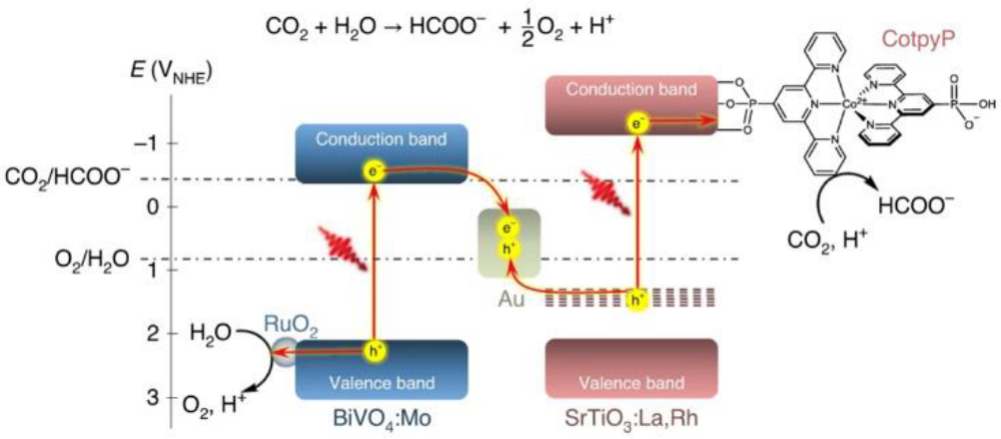
(a) An Energy Diagram Depicting the Photosynthetic CO_2_RR Coupled with Water Oxidation. The Reduction Potentials Are Given versus the NHE at pH 6.7. Reproduced with Permission from Ref (44). Copyright 2020 Springer Nature Publishing AG

It should be noted that the selective conversion of captured CO_2_ toward formic acid has been identified as one of the relatively profitable processes, although the market demand and the energy content are low (Scheme 2). Formate might be the most accessible product as all three binding modes can possibly lead to formate production (Scheme 5). Moreover, it only requires two electrons and one proton to form formate, and the breaking of the C–O bond does not occur during the formation of HCOOH. So far, the benchmark efficiency in photocatalytic CO_2_ reduction was achieved in a formate production system reaching a solar-to-formate conversion efficiency of 0.08 ± 0.01% on a Z-scheme system of SrTiO_3_:La,Rh/Au/BiVO_4_:Mo modified with molecular cocatalysts (Scheme 6).^44^

HCOOH has been previously proposed as an intermediate in the formaldehyde pathway via the surface adsorption involving O, including bidentate and monodentate O coordination or the mixed C–O coordination.^45^ EPR studies by Shkrob et al. demonstrated that only when CO_2_^–^ radical is doubly bound through its oxygens to the metal ions at the surface can this radical be further reduced to formate. Otherwise, the reduction is stalled.^46^ The bidentate binding of the CO_2_ via both O atoms results in the proton attaching to the C atom in CO_2_^–^ to form the formate HCOO radical, which further accepts one electron and proton to form HCOOH, being a favored reaction in water with a high dielectric constant.^47,48^ Regarding the further reduction of formic acid, Koci et al. showed the profiles of produced CH_4_ and CH_3_OH disagreed with the formaldehyde pathway, as CH_3_OH is not observed as an intermediate of CH_4_.^49^ Hori et al. also suggested that formate is more likely the terminal product for electrochemical CO_2_ reduction on copper except for systems with very high concentrations, high cathodic bias, and highly acidic or basic electrolytes.^50^ Therefore, we did not wholly draw the proposed debatable formaldehyde pathway (gray dashed lines) in Scheme 5. Instead, we summarize a new formyl pathway from the literature discussed in detail below.

#### Four-Electron Reduction Process

The four-electron reduction process produces HCHO and C on the basis of eqs 6–9, while HCHO production requires water oxidation (eq 1).
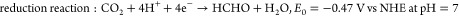
6
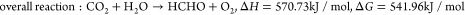
7
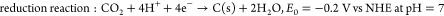
8
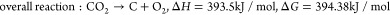
9

Formaldehyde is helpful for disinfection and is a precursor to more complex compounds in industry. In the proposed formyl pathway (Scheme 5), if the catalyst has moderate adsorption strength, the C-anchored CO intermediate can accept one electron while a proton attacks the C atom, forming the formyl intermediate (CHO), as shown in Scheme 5, which could further be converted to CH_2_O with the other proton–electron pair.^39^ By calculation of Gibbs free energy differences and the energy barriers, Cheng et al. showed that adding the proton to C (forming CHO) is preferable to adding to O (forming COH) on Cu (100), which leads to the reaction toward the formyl pathway over the carbene pathway.^51^ Formaldehyde could then be formed after receiving another pair of electron and proton. If the formaldehyde is not desorbed, the path further leads to CH_*x*_ species that can produce methane, as observed experimentally.^45^ Cheng et al. also calculated that although CH_2_O has been detected as a product, the energy barrier of CH_2_O formation is 0.47 eV higher than for CHOH and hence is kinetically unfavorable.^51^ In other words, reactions on Cu (100) should more likely have CHOH as an intermediate. CH_2_O is detectable as a product only if it is not strongly chemically bonded to the oxide surface. Nie et al. suggested that the COH intermediate more likely exists on Cu (111), since the CHO intermediate needs to overcome a significant energy barrier, blocking further reactions to produce CH_3_OH and CH_4_ by calculation study.^52^ Therefore, it is concluded that the exposed surface facets dominate the products and pathways.

**Scheme 7:**
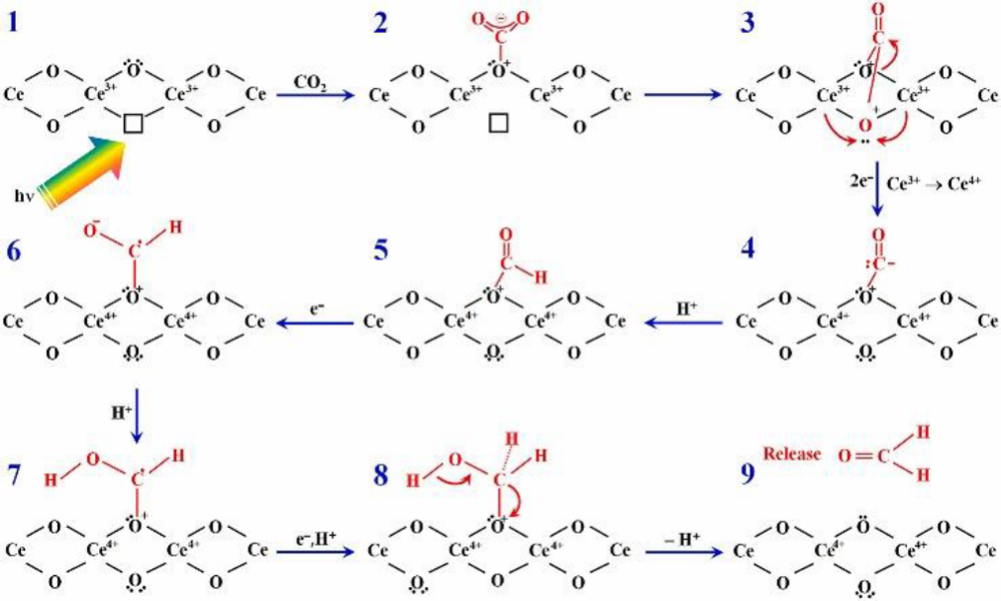
Photocatalytic CO_2_ Reduction Process on NiO/CeO_2_/rGO Hybrid Composite Photocatalyst Reproduced with Permission from Ref (53). Copyright 2021 Elsevier Reaction mechanism and pathways associated to photoactivation of CO_2_ molecules at oxygen vacancy sites of CeO_2_ surface and CO_2_ reduction through proton-coupled electron transfer processes at different stages of reaction.

However, Park et al. proposed an oxygen vacancy mediated mechanism to release formaldehyde on an rGO-grafted NiO-CeO_2_ sample (Scheme 7).^53^ The CeO_2_ surface and CO_2_^•–^ radicals were monitored by *in situ* X-ray absorption near edge structure (XANES), *in situ* attenuated total reflectance infrared (ATR-IR) and *in situ* EPR, and then a detailed description of the multistep CO_2_ photoreduction process was illustrated. Briefly, CO_2_ capture occurs at the oxygen site adjacent to oxygen vacancy. One O atom from the CO_2_ molecule fills the vacancy site, and the C–O bond is broken. The formation of CHO, CHOH, and CH_2_OH occurs through a few electron and proton transfer processes, and then formaldehyde is produced. The formation of oxygen vacancies is due to solar light illumination, which could be validated by *in situ* XANES spectroscopy. Hence the catalytic process is sustainable, and this also indicates that a combination of these characterization techniques can reveal the mechanism in a straightforward way.

Carbon could also be formed as a four-electron product in the carbene pathway. When the proton attacks the O atom of the adsorbed CO followed by eliminating one OH^–^ (or with another proton to eliminate one H_2_O), carbon radicals are generated (Scheme 5).^14^ Such a process has been verified by the signals of the C residue on the surface detected by EPR spectroscopy.^37^ DeWulf et al. also observed graphitic carbon species by X-ray photoelectron spectroscopy (XPS) and Auger electron spectroscopy (AES).^54^ Such surface carbon product or intermediate could further be reduced to CH, CH_2_, and CH_3_ over relatively low barriers.

Besides, three theoretical models have been proposed to investigate the CO_2_ reduction process via the formyl pathway, including implicit solvent models, explicit solvent models, and the H-shutting model.^16^ In the implicit solvent model, a continuous description of the ions is included, whereas the explicit solvent model offers an atomistic-level picture of solvation and cation effects.^55^ Furthermore, the H-shuttling model considers that water molecules shuttle the protons, and CHO radicals are formed via a direct transfer of H.^56^ However, on the Cu surface, explicit calculations concluded that all steps after the formation of CO radicals are proton–electron transfers. The inconsistencies in the explanation for CO_2_ reduction pathways come from both different study methods and the intrinsic complexity of the reaction mechanism. Clearly, more efforts are required to clarify this ambiguous pathway, and the combination of state-of-the-art operando spectroscopies can provide a strong potential to acquire the solution to this.

#### Six-Electron Reduction Process

The six-electron reduction process produces CH_3_OH on the basis of eqs 10 and 11, together with water oxidation (eq 1).
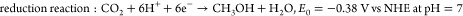
10
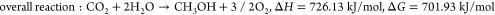
11

In the suggested “methanol economy” by Olah et al., methanol could be a crucial alternative to fossil fuels as it is a suitable energy-storage material, a fuel, and a feedstock to synthesize hydrocarbons and their products.^57^ In the context of the “hydrogen economy”, methanol is also regarded as a promising liquid medium to store hydrogen safely and efficiently before use.^58^ Methanol contains 40% more hydrogen mass density (kg H_2_ per m^3^) as well as over 80% more volumetric energy density than liquid H_2_. In contrast, the energy for compression and liquefaction of H_2_ accounts for 10–15% and 30–40% of the energy contained, respectively.^59−61^ Methanol also has a suitable balance between market demand, market price, and energy content (Scheme 2). Therefore, photocatalytic CO_2_ conversion by water to methanol with a high selectivity has attracted substantial attention. The reduction potential (−0.38 V vs NHE at pH = 7) is slightly more positive than proton reduction (−0.41 V vs NHE at pH = 7); hence, a large group of photocatalysts reported for hydrogen production could be applied. However, the 6-electron process of methanol generation requires an exceptionally prolonged lifetime of charge carriers for accumulation. Moreover, the oxidation of methanol with holes (∼10 ns) on TiO_2_ is kinetically much more favored over water oxidation (up to ∼1 s), making the continuous production of methanol with high select ivity a significant challenge.^42^

In the carbene pathway, the C radicals continue to accept three electron–proton pairs, forming the methyl CH_3_ radicals. Methanol could then be produced if the methyl CH_3_ radical recombines with a hydroxyl radical (^•^OH).^14^ In this case, methanol is not intermediate to CH_4_, and formaldehyde was not formed at all. Electrochemical studies also support that methanol could not be reduced to form methane.^54,56^ Instead, CH_3_OH is a competitive product to CH_4_, depending on a few factors such as the hydrophilicity of the surface and water amount.^62,63^ The kinetic model of such a mechanism agrees with the experiment results of methanol and methane production reported by Tan et al. and Koci et al. based on TiO_2_.^38,49^ CO is commonly observed in experiments with methanol as the major product since CO is an intermediate in this mechanism.

In another C-coordinated formyl pathway, the adsorbed CO was not dehydrated to C radicals but was attacked by electron–proton pairs. Interestingly, despite the variety of proposed paths how the electron and protons are bought to the CO radical intermediate, namely, CHO/COH or CHOH/CH_2_O, it seems that they eventually all lead to CH_2_OH and CH_3_O, as shown in Scheme 5, which possibly desorb as a CH_3_OH, although not all the intermediates are evidenced by experiments.^16,29^ Lum et al. carried out an electroreduction experiment of C^16^O_2_ in H_2_^18^O on various Cu surfaces to identify the mechanism and found that CH_3_OH was not ^18^O rich, and it was only detected on Cu (111).^64^ This interesting experiment shows the face-dependent selectivity of CO_2_ reduction and, more importantly, indicates that the O in CH_3_OH comes from CO_2_ at the beginning instead of water. This result suggests that the formyl pathway or glyoxal pathway is more likely for methanol production than the carbene pathway, where OH radicals might come from water. Cheng et al. reported that at low pH, the intermediate CHOH underwent the dehydrating process, which led to the formation of CH_3_ radicals and then a higher selectivity to CH_4_ over CH_3_OH. In contrast, a high pH, more hydrophobic environment, weak hydrogen bonding solvent, or gaseous phase benefits the selectivity toward CH_3_OH.^51^ In fact, methanol is very often a competitive product to CH_4_ in all the proposed pathways in Scheme 5, including the glyoxal pathway. It should be noted that the production of methanol is very challenging, as the final step of releasing methanol in both the carbene pathway and formyl pathway require hydroxyl radicals, and the glyoxal pathway involves photoholes, both of which are highly oxidative. The upper routine to methanol in the formyl pathway (Scheme 5), where the intermediates are continuously bonded via carbon to avoid the oxidation of intermediates, prefers to produce methanol with high selectivity.

**Scheme 8:**
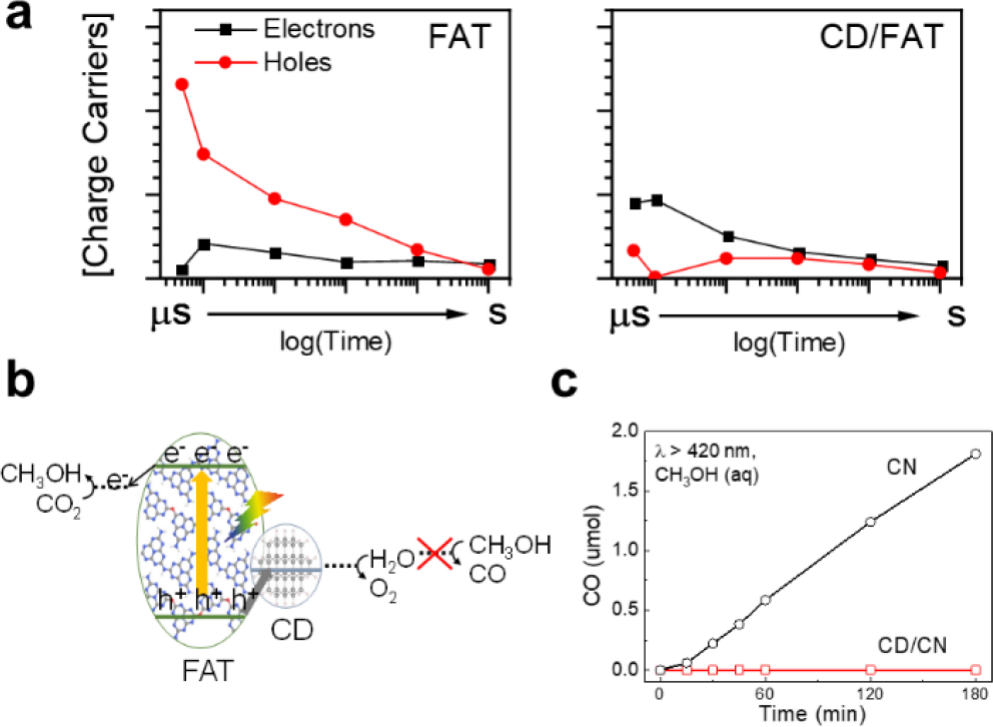
(a) Charge Carrier Populations of Electrons and Holes Determined from Spectral Deconvolution of the TAS Spectra for FAT, CD/FAT, CN, and CD/CN samples. (b) Schematic Diagram of Photocatalytic CO_2_ Reduction by the CD/FAT. (c) Methanol Oxidation Tests in the Presence of Light and CN, ^m^CD/CN Catalysts. Reproduced with Permission from Refs (42, 43). Copyright 2020 Springer Nature Publishing AG. Copyright 2021 Wiley-VCH

The stable production of methanol not only relies on the sufficient transport of electron–proton pairs but also depends on whether the produced methanol could be protected from the strongly oxidative nature of photoholes. The backward reaction of methanol oxidation could be determined by the CO yield in the products. A strategy to avoid such an issue is to suppress the adsorption of the methanol on these oxidation sites and to promote the kinetically sluggish oxidation of water against methanol. One can know whether the holes oxidize water or methanol by measuring stoichiometry between reduction and oxidation products during CO_2_ reduction. We have recently reported a unique hole-accepting carbon-dots cocatalyst (CD), where water instead of methanol selectively adsorbs (Scheme 8).^42^ Such CD prolongs the lifetime of charge carriers on pristine and oxygen-doped carbon nitride photocatalysts by 6–8 folds, resulting in the almost unity selectivity to methanol with stoichiometric oxygen production and internal quantum yields (IQYs) of 2–6% at 420 nm.^43,65^ A transient absorption spectroscopic investigation shows that extraction of holes by the CD is critical for the remarkable performance. Under visible light, methanol can be readily oxidized to CO by photoholes of carbon nitride (CN), while on the CD-decorated CN, no CO was detected, consistent with that methanol could not be readily adsorbed on the surface of CD where holes accumulate, and photooxidation of water occurs.

#### Eight-Electron Reduction Process

The eight-electron reduction process produces CH_4_ on the basis of eqs 12 and 13, together with water oxidation (eq 1).
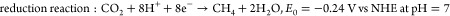
12
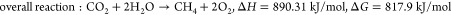
13

Methane is not only fuel but also a precursor for syngas, hydrogen, and methanol via its reforming.^66^ The methane production requires the least negative reduction potential but the most significant amount of electrons in C_1_ products. As discussed above, the carbene pathway for methane formation is more plausible than the formaldehyde pathway (Scheme 5) via the acceptance of four electron–proton pairs on the C radicals (Scheme 5).^14^ In such a case, CO is an intermediate, and methanol is a competitive product. A low pH in an aqueous solution promotes the selectivity toward CH_4_, which could also be beneficial via the modification of surface hydrophilicity by deposition of, for example, Pt NPs.^51^ In the formyl pathway toward CH_3_OH, the CH_3_O intermediate is found using the explicit water model not to form CH_4_ because of the significant barrier for proton–electron transfer from CH_3_O to CH_4_ + O.^36^ The protonation of *CO to *CHO is the rate-determining step, which is followed by a series of proton–electron transfers to form *CHOH, *CH, *CH_2_, *CH_3_, and finally CH_4_.^36^

**Scheme 9:**
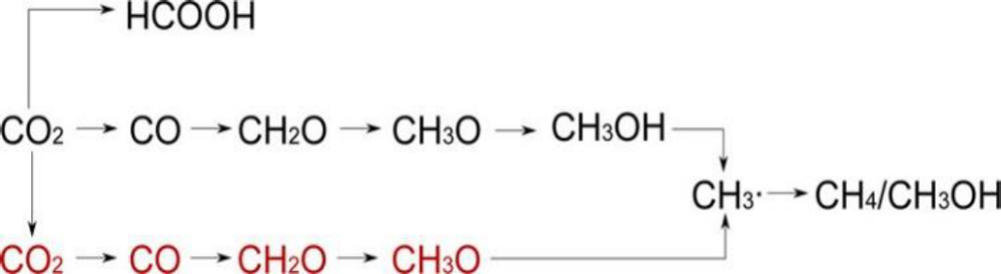
Mechanism for the Photoreduction of CO_2_ on the Perfect (Black) and Defective (Red) Surfaces. Reproduced from ref (67). Copyright 2016 American Chemical Society

Surface oxygen vacancies are commonly used strategies to modify a photocatalyst. Ji et al. proposed a mechanism for the perfect and defective surfaces by theoretical calculation (Scheme 9). They found that a fast-hydrogenation path can occur at both the surface Ti atoms and the oxygen vacancies, while the oxygen vacancies are more active than the surface Ti atoms for the reaction. The fast-hydrogenation path at the oxygen vacancies combines the hydrogenation and deoxygenation paths.^67^ This pathway agrees with the experimental results because CH_3_OH and CH_4_ often appear simultaneously and explain the possible formation of formic acid and formaldehyde.

**Scheme 10:**
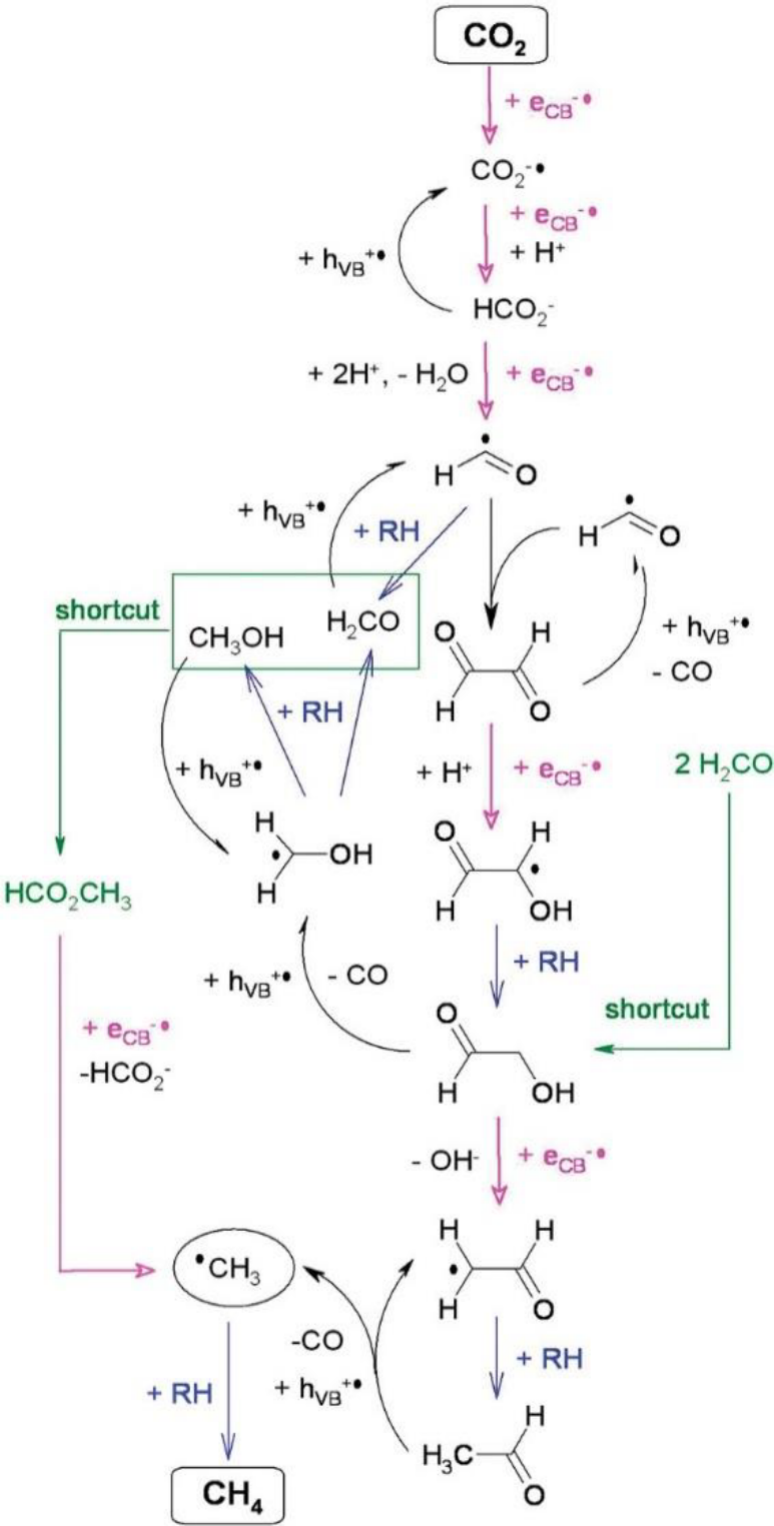
“Glyoxal Cycle”. Reproduced from Ref (41). Copyright 2012 American Chemical Society In this scheme, RH stands for the generic (molecular or radical) donor of H atoms.

As discussed above, the existence of more active intermediates, including formaldehyde and methanol in the presence of highly oxidative photoholes, contradicts the formaldehyde pathway for CH_4_ formation, in that one-electron reduction of these molecules in the reaction is energetically prohibitive.^39^ Shkrob et al. found that reaction barriers for further reduction could be significantly lowered by joining two carbon atoms and proposed to follow the glyoxal (CHO)_2_ pathway toward CH_4_ involving a number of C_2_ compounds (Scheme 10). In this pathway, the critical intermediate is the glyoxal (or ethanediol), produced from the dimerization of formyl radicals CHO radicals (Scheme 5).^41^ Because of its π-conjugation, glyoxal is still a much more efficient electron acceptor than formaldehyde, which can be step-by-step reduced to glycolaldehyde (HOCHCHO), acetaldehyde (CH_2_CHO), and acetaldehyde. These C_2_ molecules can be further oxidized to an unstable acetyl radical, which undergoes decarboxylation to a methyl radical. The recombination of the methyl radical with a hydrogen atom forms CH_4_. Formate, methanol, and formaldehyde are intermediates all formed in this pathway and serve as sacrificial hole scavengers. The glyoxal pathway will be further discussed later.

The representative examples of photocatalytic systems of CO_2_ reduction to C_1_ products have been summarized in Table 1. Considering the economic potential of the C_1_ products such as formate/formic acid and methanol, the efficient systems for these products are of particular interest. For example, the highest solar-to-fuel efficiency of 0.08 ± 0.01% (solar-to-formate) measured under the solar simulator was achieved on the molecular cocatalyst coated on a Z-scheme particulate sheet (phosphonated Co(II) bis(terpyridine) and RuO_2_ catalysts modified SrTiO_3_: La, Rh|Au| BiVO_4_: Mo).^44^ The benchmark system for CO_2_ photoreduction to formic acid was based on a gas-permeable metal–organic framework (MOF) with an apparent quantum efficiency (AQE) of 15.76% at 420 nm, at the porous gas–solid interfaces with a near-unity selectivity.^68^ Such a high quantum efficiency is also the highest among most systems for the C_1_ products, suggesting that the metal–organic complexes play a significant role in CO_2_ reduction. More examples of metal complex photocatalysts could be found in other reviews.^26^ The highest internal quantum efficiency at 420 nm toward methanol was measured on carbon dots/oxygen-doped carbon nitride.^43^ The key to maintaining a reasonable efficiency of methanol is to mitigate overoxidation of methanol product to CO.

**Table 1:** Representative Examples for Selective Photocatalytic CO_2_ Reduction to C_1_ Products

final product	electron transfer	catalyst	production rate	pressure and temperature	other conditions	selectivity	efficiency
CO	2	Au-MMT/TiO_2_^69^	1223 μmol g^–1^ h^–1^	atm.; 100 °C	CO_2_ and H_2_, UV-light	99%	\
		Ru@Co_3_O_4_^70^	2003 μmol g^–1^ h^–1^	1 atm; 15 °C	30 mL, acetonitrile/TEOA/H_2_O = 3:1:1 (v/v), 300 W xenon lamp with a 420 nm cutoff filter	77%	EQE = 0.069%
		Ru@g-C_3_N_4_^71^	181.25 μmol g^–1^ h^–1^	\	400W Hg lamp, 20 vol % TEOA in DMA,	64%	\
		TiO_2_/CsPbBr_3_^72^	9.02 μmol g^–1^ h^–1^	80 kPa; 10 °C	30 mL of acetonitrile, and 100 μL of water; 300 W Xe arc lamp	95%	\
HCOOH	2	photocatalyst sheet SrTiO_3_: La, Rh|Au|RuO_2_–BiVO_4_: Mo^44^	1.09 ± 0.12 μmol cm^–2^ h^–1^	atm.	CO_2_ saturated 0.1 M KHCO_3_	97 ± 3%	solar-to-formate conversion efficiency of 0.08 ± 0.01%. AQY = 2.6% (420 ± 15 nm)
		Ru@g-C_3_N_4_^73^	46.425 μmol g^–1^ h^–1^	r.t.	λ> 400 nm, 20 vol % TEOA in acetonitrile	\	\
		single metal atom @ A-aUiO^68^	3.38 mmol g^–1^ h^–1^	\	particle-in-solution mode, and gas-membrane-gas mode	near unity	AQE = 2.51% (gas–liquid–solid reaction interface); AQE of 15.76% (porous gas–solid interfaces, 420 nm)
HCHO	4	Pt and Cu @K_2_Ti_6_O_13_ particles^74^	3.42 μmol g^–1^ h^–1^	r.t.	300 W Xe lamp; H_2_O and CO_2_	\	\
		Au NPs @TiO_2_ films^75^	1.36 μmol g^–1^ h^–1^	\	254 nm UV	\	\
		NiO/CeO_2_/rGO^53^	421.09 μmol g^–1^ h^–1^	\	0.2 M NaHCO_3_ and 0.2 M Na_2_SO_3_ solution	\	solar to fuel conversion efficiency 0.775%
C	4	reduced NiFe_2_O_4_^76^	\	atm.; 80 °C	catalyst was uniformly dispersed onto the quartz glass reactor	\	\
CH_3_OH	6	carbon dot/C_3_N_4_^42^	13.9 ± 1.7 μmol g^–1^ h^–1^	atm.; r.t.	H_2_O and CO_2_	99.60%	IQY: 2.1% (420 nm), 0.7% (500 nm), 0.4% (600 nm)
		carbon nitride-like polymer (FAT) decorated with carbon dots^43^	24.2 μmol g^–1^ h^–1^	atm.	H_2_O and CO_2_	100.00%	IQY: 5.9% at λ=420 nm
		carbon nitride-CdS QD^77^	186.4 μmol g^–1^ h^–1^	\	CO_2_ in 40 mL of KHCO_3_ (0.1M) and Na_2_SO_3_ (0.1M)	73%	AQE = 0.91% (435 nm)
CH_4_	8	BiVO_4_/WO_3_^78^	105 μmol g^–1^ h^–1^	\	30 mL of 0.1 M NaOH, purged with high purity CO_2_ for 45 min	99%	0.074% solar to methane efficiency
		Ni@SiO_2_-Al_2_O_3_^79^	4200 μmol g^–1^ h^–1^	150 °C	H_2_ (14.5 mmol, 80 v/v %) and CO_2_ (3.63 mmol, 20 v/v %); 300 W Xe lamp, 1 KW/m^2^	\	\
		CoDAC-3.5 with BV NSs^80^	19.5 μmol g^–1^ h^–1^	\	H_2_O and CO_2_	65%	AQY = 5.23% (400 nm)

### Pathways to C_2_ Products

The direct photoconversion of CO_2_ to C_2_ products is more attractive because of their significantly higher market price and energy content per carbon compared with the C_1_ products (except for methanol in Scheme 2).^16^ The formation of the C–C bond by dimerization of C_1_ intermediates on the surface of catalysts is the key to further producing C_2_ products. For example, the pathways toward ethylene or ethane involve the coupling of carbene intermediates (CH_2_, CH_3_) in the carbene pathway while the dimerization of formyl (CHO) in the glyoxal pathway can result in a variety of C_1_ and C_2_ products (Scheme 5). These pathways mostly require multiple electrons and protons, which will be favorable on the surface with an increased density of charge carriers and protons. Also, the binding of intermediates on the surface of the catalysts must be sufficiently strong to reach the C_2_ products instead of desorbing them early as C_1_ products such as CO and CH_2_O. C_1_ products such as CO, CH_4_, and CH_3_OH can also be byproducts of these processes. All these processes again share the water oxidation half-reaction (eq 1). The following lists the chemical eqs (eqs 14–25) for C_2_ products with 2 to 14 electrons, respectively.

#### Two-Electron Reduction Process

The two-electron reduction process produces (COOH)_2_ on the basis of eqs 14 and 15.
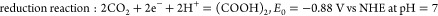
14
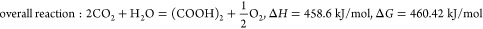
15

#### Eight-Electron Reduction Process

The eight-electron reduction process produces CH_3_COOH on the basis of eqs 16 and 17.
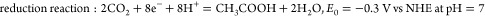
16
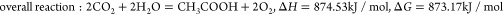
17

#### Ten-Electron Reduction Process

The 10-electron reduction process produces CH_3_CHO on the basis of eqs 18 and 19.
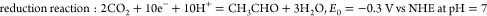
18
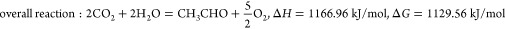
19

#### Twelve-Electron Reduction Process

The 12-electron reduction process produces CH_3_CH_2_OH and C_2_H_4_ on the basis of eqs 20–23.
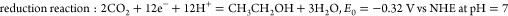
20
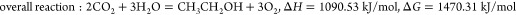
21
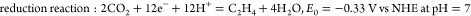
22
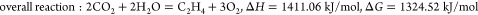
23

#### Fourteen-Electron Reduction Process

The 14-electron reduction process produces C_2_H_6_ on the basis of eqs 24 and 25.
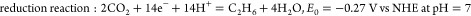
24
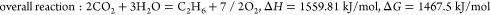
25

Since the pathways to C_2_ products are believed to involve both multi-electron coupled proton transfer and the C–C coupling processes, the reactions are generally more challenging, and fewer reports have achieved high selectivities.^81^ Different from the reactions toward C_1_ products, which are mostly proton-coupled one-carbon two-electron processes, the pathway to C_2_ products proposed by Shkrob et al., namely the glyoxal pathway (Scheme 10), is a mainly one-electron two-carbon process through a glyoxal intermediate (*e.g.*, on TiO_2_) as evidenced by EPR.^41^ The reason to postulate an alternative mechanism is that no evidence of one-electron reduction intermediate (*e.g.* formate, formaldehyde, or methanol) has been observed in EPR. As mentioned above, these intermediates are more likely to be oxidized than reduced. However, the glyoxal intermediate is a strong electron acceptor, which is more facile to be reduced than oxidized. Moreover, the glyoxal pathway can provide mechanisms for C_1_ products such as CO, CH_4_, methanol, formate and formaldehyde and explain the possible C_2_ products observed, including acetaldehyde and methylformate.

As shown in Schemes 5 and 10, different from the de–OH^–^ step after receiving one proton in the carbene pathway, the crucial step in the glyoxal pathway is the dehydration step after getting three protons (or de-OH^–^ step after accepting two protons^14^), forming formyl radicals (HC*O). Then the formyl radicals dimerize to yield glyoxal, which is reduced to trans-ethan-1,2,-semidione (HO-C*H-CHO), glycolaldehyde (HO-CH_2_-CHO), and vinoxyl (*CH_2_-CHO) radicals. The vinoxyl radical could be further converted to acetaldehyde (CH_3_CHO) or decarbonylized to *CH_2_OH, which is the precursor for methanol (CH_3_OH). The acetaldehyde could be further oxidized to acetic acid or undergo decarbonylation to form methyl radicals (*CH_3_), which leads to the formation of methane (CH_4_). These decarbonylation processes release carbon monoxide (CO). In this case, CO is a byproduct commonly observed in CH_4_ and CH_3_OH production.

The most distinct difference between the glyoxal cycle and other pathways is that the former involves not only proton-coupled electron transfer but also oxidation steps. Such pathways are not possible in the absence of holes, for example, at the cathode in the electrocatalytic system. However, the existence of holes results in the production of CO rather than C_2_ products. Therefore, if one targets value-added C_2_ products, the oxidative conditions should be mitigated. Another interesting difference is that the glyoxal pathway has some recycling steps where some of the intermediates (*e.g.*, CH_3_OH and CH_2_O) serve as hole scavengers, hence replenishing the pool of formyl and hydroxymethyl radicals. Compared with C_1_ products, glyoxal and methanol have the same required electrons with the number of 6, and the acetic acid and glycolaldehyde have the same electrons needed with the number of 8 as methane. The production of acetaldehyde and ethylene glycol need 10 electrons, while ethylene and ethanol need 12 electrons. It should be noted that the 10-electron product acetaldehyde is an intermediate for an 8-electron product CH_4_ and a 2-electron product CO, which are much cheaper. It is not economically favorable to oxidize acetaldehyde to CH_4_ and CO, considering the high numbers of demanded electrons and the low price of methane in the market. It is preferable to produce the C_2_ products with those electrons. How CO_2_ molecules bind on the surface of the catalysts usually determines the following steps for various possible products. In the pathways to C_2_ products, C–C, C–O and single C bindings probably exist at the initial stages when forming glyoxal intermediates, and the coordination might rearrange to O–O binding during the reaction. However, it seems that a few binding modes and the following steps all can lead to products including glycolaldehyde, acetaldehyde, ethylene glycol, ethylene, and ethanol.^82^

**Scheme 11:**
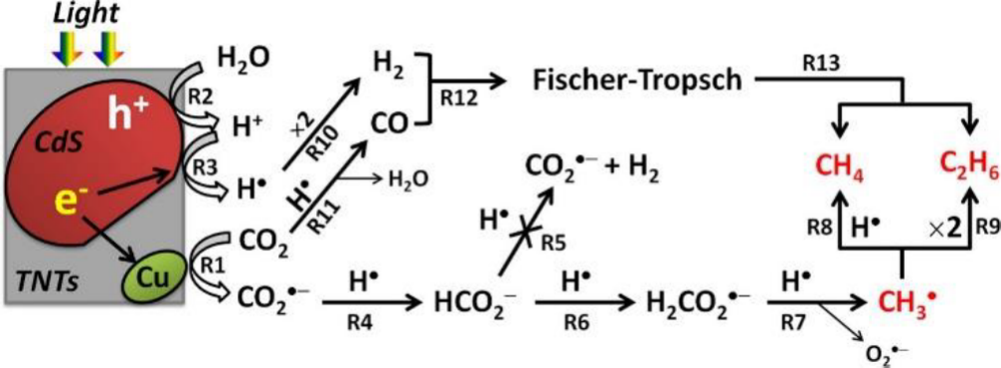
Proposed Elementary Reaction Pathways of Photocatalytic CO_2_ Conversion into Hydrocarbons. Reproduced from Ref (83). Copyright 2015 American Chemical Society

Park et al. reported a CdS/(Cu-TNT: Na_*x*_H_2–*x*_Ti_3_O_7_) for photoconversion of CO_2_ and water into C_1_–C_3_ hydrocarbons (*e.g.*, CH_4_, C_2_H_6_, C_3_H_8_, C_2_H_4_, C_3_H_6_) under visible light without the detection of CO or H_2_ (Scheme 11).^83^ It could go through an F–T process consuming hydrogen and CO. They also proposed that the bidentate binding of O=C=O to specific reactive surface sites reduced the energy barrier for conduction band electron transfer to CO_2_. The formate radical eliminates an O_2_^•–^ radical, forming a methyl radical. The methyl radical (CH_3_^•^), as observed by ESR, was trapped by the copper on the surface of TNTs and then self-reacts to produce ethane.^83^ This pathway indicates that the C–C coupling of methyl radicals on the surface of photocatalysts is crucial for the production of C_2+_ products such as ethane.

Regarding the C_2_H_6_ formation on the surface of Pt-graphene/defect-induced TiO_2_, where CO_2_ adsorption takes place upon abundant Ti^3+^/V_O_ sites through the O atom of CO_2_.^84^ An alkaline surface is favorable for C_2_H_6_ formation by significantly improving the activation and dissociation.^85^ Electrons and holes accumulate on Ti^3+^ and graphene, respectively, with Pt accelerating electron extraction. Graphene scavenges water to supply sustained protons and benefits the stabilization of ^•^CH_3_, thus promoting C_2_H_6_ formation, although CH_4_ is also a major product.

Hori et al. and Schouten et al. showed that Cu (111) leads to lower onset overpotentials for both CH_4_ formation and C_2_H_4_ formation, while the dimerization of CO leads to C_2_H_4_ as the product on Cu (100).^86,87^ The dimerization of CO is followed by a few steps which are similar to the glyoxal pathway, except that the intermediate (CH_2_CHO) changes from C-coordination to O-coordination. CH_2_CHO^•^ could further produce C_2_H_4_ or CH_3_CH_2_OH.^45^ It should be noted that these reports are based on studies on the electrochemical reduction of CO_2_, and the pathways to C_2_ products have not been observed in photocatalytic research.

There is also a possibility that C_2_ products can be produced via the carbene pathway (Scheme 5). In this pathway, another carbene radical might attack the formed CH_4_ or CH_3_OH to make C_2_H_4_ or C_2_H_6_.^31^ Other products, including C_3_ and C_3+_, could also be obtained if more carbene radicals are involved in such a mechanism but are hardly reported.^31^ Kuhl et al. have proposed an enol-like intermediates pathway. However, how the enol (*e.g.*, C_2_H_4_O_3_, C_3_H_6_O_2_) is formed on the surface of catalysts remains unclear.^88^ Strategies that increase the density of charge carriers and protons, tune the surface adsorption/desorption, and modify the surface acidity and alkalinity are believed to promote the reaction toward C_2+_ products, including using a larger number of photon flux and higher energy, cocatalysts, SPR effects and surface defects.^17^

Most of the C_2_ products are valuable products. As summarized in Table 2, the benchmark quantum efficiency for C_2_ products is 22.4% at 385 nm and 13.3% at 420 nm (CO_2_ to acetaldehyde with a 98.3% selectivity) achieved on locally crystallized carbon nitride.^89^ Liu et al. used DRIFT and theoretical calculation to prove that an amino-2-propanol-assisted hydrothermal treated carbon nitride had strong bonding with the *OCCHO group, which is favorable to a C–C coupling process and changes the reaction pathway to form CH_3_CHO instead of HCHO. The highest quantum efficiency for ethanol was 3.5% on AgBr–N-doped carbon nitride. While the activity and selectivity for ethylene and ethane are generally much smaller, which might result from the large numbers of electrons needed for these products (12–14 electrons).

**Table 2:** Representative Examples for Selective Photocatalytic CO_2_ Reduction to C_2_ products

final product	electron transfer	catalyst	production rate	pressure and temperature	other conditions	selectivity	efficiency
CH_3_COOH	8	partially reduced Co_3_O_4_ nanosheets^90^	3 μmol g^–1^ h^–1^	25 °C	simulated air (0.03% CO_2_)	92.50%	2.75% CO_2_-to-CH_3_COOH conversion ratio
		metal–organic framework/MoS_2_^91^	39.0 μmol g^–1^ h^–1^	0.1 MPa	H_2_O and CO_2_ with visible light	94.00%	\
		1%MgO@TiO_2_^92^	1.04 μmol L^–1^ g^–1^ h^–1^	25 °C	100 mL 0.1 M NaOH with CO_2_ ; 253.7 nm (0.167 mW/cm2)	\	\
		WO_3_·0.33H_2_O^93^	9.4 μmol g^–1^ h^–1^	4 °C	H_2_O and CO_2_ (50% in Ar)	85%	\
CH_3_CHO	10	locally crystallized polymeric carbon nitride (PCN)^89^	1814.7 μmol g^–1^ h^–1^	10 °C	3 mL of 10% triethanolamine (TEOA)/acetonitrile (MeCN) solution with 0.3 mL of H_2_O in a 60 mL sealed flat quartz reactor	98%	quantum efficiency of 22.4% at 385 nm; QE of 13.3% at 420 nm
		bulk g-C_3_N_4_^94^	0.4 μmol h^–1^	0.06 MPa	water vapor and CO_2_ UV–vis light source	\	\
		C-SnS_2_^95^	139 μmol g^–1^ h^–1^	Atm.; 25 °C	300 W halogen lamp, gas flow reactor		photochemical quantum efficiency: 0.7%
		Nb-doped TiO_2_^96^	572 μmol g^–1^ h^–1^	R.t.	NaHCO_3_ solution (0.1 M, 45 mL) with triethanolamine (5 mL) as the sacrificial agent.	98%	\
CH_3_CH_2_OH	12	red phosphorus decorated Bi_2_MoO_6_^97^	51.8 μmol g^–1^ h^–1^	\	water vapor	\	\
		AgBr–Nitrogen doped graphene–g-C_3_N_4_^98^	51 μmol g^–1^ h^–1^	r.t.	NaHCO_3_ and CO_2_, Xe lamp (450W)	\	AQY = 3.5%
		conducting polymers modified Bi_2_WO_6_ microspheres^99^	5.13 μmol g^–1^ h^–1^	4 °C	H_2_O and CO_2_	\	AQE = 0.0086% of PTh/Bi_2_WO_6_
		Bi_2_MoO_6_ quantum dots in situ grown on reduced graphene^100^	14.37 μmol g^–1^ h^–1^	4 °C, atm.	H_2_O and CO_2_	\	AQE = 0.00793% at 300 W Xe lamp with a 420 ± 15 nm band-pass filter
C_2_H_4_	12	C/Cu_2_O mesoporous nanorods^101^	0.016 μmol h^–1^	atm.; r.t.	0.1 M KHCO_3_ and CO_2_	56.1%	AQE= 1.36% at 400 nm
		Cu/TiO_2_ powders^102^	0.542 μL g^–1^ h^–1^	28 kgf/cm pressured CO_2_	H_2_O and CO_2_	48.7%	\
		g-MWCNT@TiO_2_ core–shell nanocomposites^103^	0.048 μmol g^–1^ h^–1^	bubbling CO_2_ in H_2_O at 60 °C, Atm.	H_2_O and CO_2_	5%	\
C_2_H_6_	14	CdS/(Cu-TNTs)^83^	17 μL g^–1^ h^–1^	20–25 °C and atm.	H_2_O and CO_2_, 450 W Xe lamp, 420 nm	\	\
		Nafion/Pd-TiO_2_^104^	1 μmol h^–1^	atm.; r.t.	NaHCO_3_ aqueous dispersion CO_2_ saturated, pH 1, 300 W Xe lamp (λ > 300 nm), 5 h	6.4%	\
		Pt-G/defect-induced TiO_2_^84^	11 μmol g^–1^ h^–1^	atm.; r.t.	sun simulated light (100 mW/cm^2^), moist CO_2_, continuous flow 1 mL min^–1^	22.9%	AQE = 2.7%
		AuPd/(101) TiO_2_^105^	1 μmol g^–1^ h^–1^	0.26 MPa, 40 °C	NaHCO_3_ aqueous dispersion CO_2_ saturated, 300 W Xe lamp	7%	\

## 3 CHALLENGES AND OUTLOOK

**Scheme 12:**
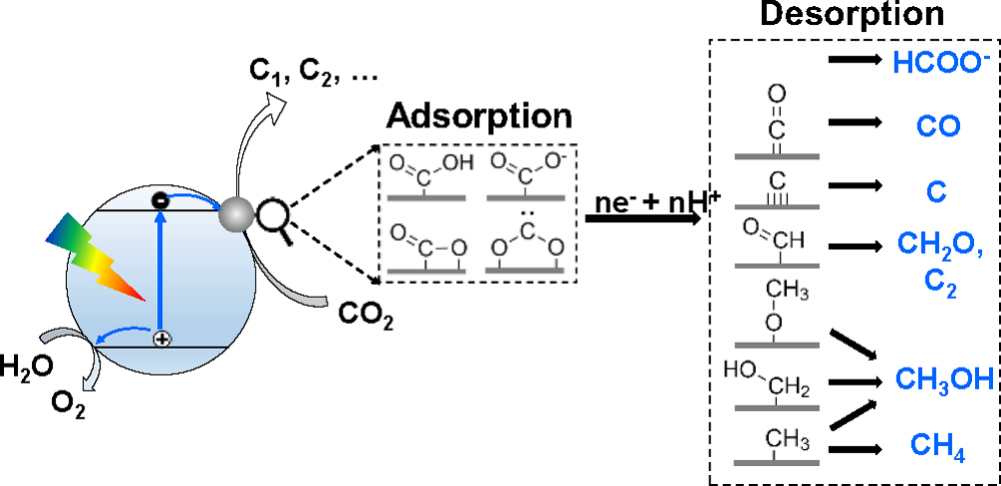
Possible Adsorption of CO_2_ and Desorption of Intermediates on the Surface of Cocatalysts in Photocatalytic CO_2_ Reduction

We have discussed the detailed potential pathways toward various products, including the well-established carbene pathway, glyoxal pathway and a formyl pathway. The reaction pathway and consequently the product selectivity of photocatalytic CO_2_ conversion highly depends on the surface chemistry of adsorption of CO_2_ and desorption of intermediates on the surface of photo/cocatalysts, which has been thoroughly summarized in other reviews (Scheme 12).^17,20,29,31,49,106^ Other factors including crystal facets, surface poisoning phenomena, which are closely related with the bonding modes and strength as discussed above, could also influence the reactivity and selectivity.^16,70,82^

The key facts of these pathways are summarized in Table 3 regarding different profiles of product distribution. For example, formate can be a promising product because it is both economically beneficial and kinetically easy to produce. However, CO exists in all the pathways. Based on the amount and production profile, the possible mechanism could be inferred. For example, if the amount of CO is minor compared with other main products, it is likely an intermediate or an oxidation product from, for example, methanol. If the amount of CO is comparable to methanol or methane, the reaction is most likely via the glyoxal pathway. Methanol and methane are always competitive products in these pathways, but methanol is more desired in the aspect of market price and transportation, providing its further oxidation could be mitigated. However, this is challenging because the final step of releasing methanol in a few pathways involves photoholes or hydroxyl radicals, which are highly oxidative. Methane requires multi-electrons (8 e^–^), but its price is even below the cost of capturing CO_2_. If the reaction goes through the glyoxal pathway, terminating the products at C_2_ before the decarbonlization is more meaningful, rather than the decomposition of the C_2_ to unprofitable CO and CH_4_. Another pathway to C_2+_ products via carbene coupling is promising as it has the potential to produce long-chain products.

**Table 3:** Summary of Important Facts for the Diverse Products from Photocatalytic CO_2_ Conversion by Three Proposed Pathways

products	remarks	carbene pathway	formyl pathway	glyoxal pathway
HCOO^–^	high market price, one-step final product, no C–O breaking, accessible from various binding modes	×	×	×
CO	uneconomic if produced with H_2_, while valuable if pure CO, may exist in all pathways	intermediate and final product if weak binding	intermediate, byproduct from *e.g.* methanol oxidation	intermediate, final byproduct to methanol or methane
C	uneconomic	intermediate and product	×	×
CH_2_O		×	intermediate and product	intermediate and product
CH_3_OH	high energy content, valuable liquid fuel	the final competitive product to methane	the final competitive product to methane	the final competitive product to methane, release with CO
CH_4_	high energy content but Uneconomic	the final competitive product to methanol	the final competitive product to methanol	the final competitive product to methanol, release with CO
C_2_H_4_, C_2_H_6_	high energy content and high market price	product from carbene coupling	×	×
C_2_H_2_O_2_, C_2_H_4_O_2_, C_2_H_4_O	high energy content and high market price	×	×	intermediate and final product if not further decarbonlised

Although substantial progress has been made in developing and understanding photocatalysts for solar fuel production from CO_2_ and water, we believe there are at least four significant challenges to overcome in this field apart from the discovery of catalysts/cocatalysts, which have been extensively reviewed by others.^16,17,27,29,31,106,107^

### Validation of the Reactions

The first challenge is the confirmation of the products in CO_2_ reduction reactions. In two studies, Yang et al. and Yui et al. observed ^12^C products (^12^CO and ^12^CH_4_, respectively) even if ^13^CO_2_ was used as the carbon source, indicating the carbon residues in the system could participate in the photoreactions hence should not be ignored. Therefore, the detection of all carbon-based products is not indeed proof of CO_2_ reduction before the validation by control experiments. Control experiments in the CO_2_ atmosphere and inert gas (*e.g.*, Argon) might give some hints. While the direct evidence is the isotopic labeling experiments, which could be carried out in a GCMS,^42^ an IR system^108^ or NMR^44^ to distinguish the ^13^C labeled products. Hence it is crucial for all CO_2_ reduction reactions.

Meanwhile, the oxidation products (*e.g.* oxygen) should also be scrutinized and calculated with the detected carbon products to verify whether the stoichiometry of proposed reactions is achieved. Besides, the performance reported by different groups critically depends on many specific details of the experimental setup and reaction conditions, such as the light source, the pH, cocatalyst selection and loading, the sample concentration, and the reactor design. To minimize such influences, the quantum yield (QY) together with the product generation rate is all required.

### Mechanism to Generate High-Value-Added Products

The second challenge is to understand the production mechanism of high-value chemicals, especially C_2+_, via multi-electron processes since interfacial reactions such as CO_2_ and multiproton reduction usually occur on a time scale of microseconds or longer.^28^ As observed for CN_*x*_H_*y*_, such trapping leads to a severe loss of driving force and microsecond charge transfer rate.^109^ To obtain a sufficient charge carrier lifetime without losing too much driving force for the interface redox reaction, nature uses a dual photocatalyst system instead of a single photocatalyst, allowing the realization of effective charge separation via a series of downhill charge transfers and overcome the inevitable back reaction.^110^ There were reports on the Z-scheme CO_2_ reduction systems, in which electrons and holes are generated on the spatially separated subsystems, thereby reducing the tendency of electron–hole recombination and allowing a longer lifetime and more significant accumulation of charge carriers to overcome the kinetic limitations.^44^ The accurate determination of the time scale of the charge carrier relaxation and the excited state as well as the respective reaction intermediates at the surface will provide us with the information needed to design effective materials for the selective production of the high-value products. This information can be obtained by *in situ* spectroscopies such as IR, EPR, XPE, XANES, and theoretical calculation.

### Practical Devices

The third challenge is to develop practical devices for photocatalytic CO_2_ conversion. For simplicity and to focus on evaluating materials, a closed batch reactor is the most commonly used system in the literature despite its limitations. However, for potential large-scale use in the future, rationally designed reactors such as a flow system would be beneficial, in which the light distribution and mass flow need to be considered carefully.^111^ Only limited types of CO_2_ conversion devices have been reported so far. Wang et al. reported a wireless and stand-alone photocatalyst sheet device for scalable solar formate production from carbon dioxide and water with the solar-to-formate efficiency of ∼0.1% using a nature-mimicking Z-scheme architecture.^44^ Recently, a modular 5 kW pilot-scale solar-thermal system was demonstrated to synthesize methanol from H_2_O and CO_2_ captured directly from the ambient air operated under actual conditions.^112^ This large-scale reactor could be an exciting reference for practical CO_2_ conversion, although the device was only used in thermal catalysis so far. In the broad field of photocatalysis, a water-splitting device extended to the scale of 100 m^2^ was preliminarily validated very recently, reaching a solar to hydrogen efficiency (STH) of 0.76%,^113^ which to some extent demonstrated the safe, large-scale photocatalytic water splitting and gas collection and separation. However, the overall process was summed up as energy negative. A continuous flow reactor for scalable organosynthesis was also tested.^114^ Another group of practical reactors for CO_2_ photoreduction are the membrane reactors, which immobilize photocatalyst (nano)particles on the membrane substrates to replace a suspension.^115^ Although such systems might suffer from possible catalyst losses under long-term operation, they have the advantages such as no need to separate the catalyst from the solution and highly stable catalytic efficiency.^116^ The reported examples of such membrane reactors include C_3_N_4_ or TiO_2_ on Nafion membrane,^117,118^ TiO_2_ on carbon paper,^119^ TiO_2_ and Cu–TiO_2_ on zeolitic imidazolate framework (ZIF-8),^120^ and so on, which are good examples for the potential use in a flow system. Reactors under concentrated solar power also have been developed to obtain the high density of electron–hole pairs in the photocatalyst, and the catalyst can also be heated to high temperatures.^74,121^ We have also recently developed a flow-reactor for oxidative coupling of methane to C_2_ products and a multilayer device for methanol reforming.^8,122^ All these devices for the equivalent photocatalytic reactions might accelerate the development of efficient and scalable devices for practical CO_2_ photoconversion, especially driven by the current global concern of the critical climate change.

### Economic Considerations

The last challenge, closely connected to the third, is to take the cost of the reactant CO_2_ into account, which is a limiting factor for scaling up but is often ignored. Most reports used CO_2_ in significant excess without considering the cost of CO_2_ capture processes or the conversion efficiency of CO_2_. The approaches to directly capture CO_2_ from the air include aqueous alkali capture (cost US $97–134 tCO_2_^–1^ for 0.1 MPa CO_2_, US $116–168 tCO_2_^–1^ for 15.1 MPa CO_2_), supported amine capture (cost ∼ US $90 tCO_2_^–1^) and the disruptive solid absorption (cost ∼ US $15–50 tCO_2_^–1^).^65,123,124^ Besides, other criteria such as carbon intensity (CI, the amount of net carbon by weight emitted per unit of fuel energy consumed) and the full-cycle time should also be considered.^61^ An analysis by Nitopi et al. showed that products such as methane and CO (syngas) are not economically feasible because their market prices do not make up for the energy cost of CO_2_ capture, although these products are commonly reported in the literature.^16^ Instead, more preferable C_1_ products include methanol, formic acid, and CO (pure), while promising C_2+_ products are ethylene, ethanol, acetaldehyde, and propanol.^16^ The direct reduction of CO_2_ from the air is a very high cost and challenging process since CO_2_ only accounts for ∼400 ppm in the atmosphere, and the existence of oxygen also tends to react with photoelectrons to form O_2_^–^ competitively. A preliminary example is the photocatalytic CO_2_ conversion directly from air with a ca. 98.35% selectivity to CH_3_OH and ca. 4.32% conversion efficiency of CO_2_ after 4 h reaction by selectively adsorbing CO_2_ rather than O_2_ on Rb_0.33_WO_3_ catalyst.^125^ Clearly, this is promising, while the economic benefit is too early to assess. However, the development of routes for high value-added products could be promoted by the driving force from the market.

Overall, the photocatalytic efficiency of CO_2_ to chemicals, in particular, the selectivity to the C_2_ chemicals, remains moderate so far, and its economic and environmental feasibility should be evaluated carefully to avoid extra carbon emission. An in-depth understanding of the reaction pathway will enable a precise design of the photocatalytic system, including the semiconductor, surface modification, defect engineering, cocatalyst loading, reaction conditions, and so on, toward preferable products. All these are at a very early stage at present, requiring multidisciplinary efforts and collaborations to overcome the barriers facing.
